# Ultrasound evaluation of scar thickness for prediction of uterine dehiscence in term women with previous caesarean sections

**DOI:** 10.12669/pjms.40.7.8712

**Published:** 2024-08

**Authors:** Shaista Afzal, Imrana Masroor, Ahsun Amin, Aiman Majeed

**Affiliations:** 1 Shaista Afzal, FCPS. Associate Professor, Radiology Aga Khan University, Karachi, Pakistan; 2 Imrana Masroor, FCPS. Professor, Radiology Aga Khan University, Karachi, Pakistan; 3 Ahsun Amin, MSc. Epidemiology and Biostatistics Senior Instructor Research, Radiology, Aga Khan University, Karachi, Pakistan; 4 Aiman Majeed, FCPS. Instructor, Obstetrics and Gynaecology, Aga Khan University, Karachi, Pakistan

**Keywords:** Ultrasound, Lower uterine scar thickness, Uterine dehiscence, Caesarean section

## Abstract

**Objective:**

To determine the role of ultrasound in evaluation of scar thickness for prediction of uterine dehiscence.

**Method:**

This retrospective cross-sectional study was conducted in the Radiology department of Aga Khan University Hospital from 1st July to 31st December 2021 after approval from the University Ethic Committee. In this study pregnant women 18 to 40 years with a live singleton fetus with vertex presentation, at term, with history of prior caesarean section and availability of medical record were included. Using a curvilinear ultrasound transducer with optimally distended urinary bladder, the myometrial thickness was measured in the sagittal plane. The intraoperative visual findings of the lower uterine segment outcome at the time of C-section were recorded and categorized into two groups i.e., with and without dehiscence for statistical analysis.

**Results:**

A total of 126 women were included. The mean age of the study participants was 29.8±4.1. The median gestational age was 35 (34-37) weeks. The highest AUC 0.58 was recorded for the scar thickness of ≤2.5mm with a sensitivity, specificity, PPV and NPV of 80.9%, 36.4%, 36.3% and 80.8% respectively. Similarly, the AUC for the scar thickness of ≤2mm was 0.55 with a sensitivity, specificity, PPV and NPV of 93%, 18.2%, 18.2% and 93% respectively.

**Conclusion:**

Transabdominal Sonography is a safe technique to determine the LUS thickness during antenatal ultrasound at term. A cutoff value of ≤2mm showed a high sensitivity and negative predictive value of 93% for evaluating the risk of uterine dehiscence.

## INTRODUCTION

The most commonly performed surgery in obstetrics is the Caesarean section (C-section) accounting for one third of all cases. The rate of caesarean deliveries (CD) has significantly increased from 5% in 1970 to 31.9% in 2016.^1^,^2^

The increasing frequency of C-section is attributed to electronic fetal monitoring, decreasing frequency of vaginal breech delivery and history of C-section which alone account for more than a third of cases annually.^3^ One of the many reasons that contribute to the fall of vaginal birth after caesarean (VBAC) is the possibility of uterine rupture, which can contribute to serious maternal and perinatal complications. Hence, it’s essential to determine the integrity of uterine scar ahead of vaginal delivery trial. Ultrasound (US) is an appropriate modality which can confirm the integrity of the lower uterine segment scar and can detect the defect with reasonable accuracy. The other advantages of utilizing US for this diagnosis are its quick to perform, noninvasive, no radiation exposure and can be used for patient follow up. Hence, the rationale of the study was to explore the utility of US in scar thickness measurement and its value in predicting the scar integrity in term women with previous C-section. Thus, providing information that facilitates appropriate patient management.

The long-term complications of CDs, in subsequent pregnancies particularly placenta previa and morbidly adherent placenta, are concerning as these significantly contribute to maternal and neonatal morbidity and mortality, and the risks rise with each additional CD.^4^

An important step in lowering the rate of caesarean deliveries is to encourage more women with previous caesarean delivery to try a VBAC. The complications of VBAC include caesarean scar dehiscence, leading to uterine rupture in 0.7% and potentially fatal outcomes for the mother and neonate. The strength of the scar is linked to its thickness and determines the outcome of VBAC.^5^ Uterine rupture entails direct connection between the peritoneal and amniotic cavity resulting in intra-abdominal hemorrhage and irregularity of fetal heart-rate tracing. Uterine dehiscence on the other hand refers to an asymptomatic uterine defect frequently observed during a subsequent C-section and indicate a higher risk of intrapartum uterine rupture if it is discovered prior to labour.^6^

The integrity of lower uterine scar (LUS) can be evaluated by measurement of LUS thickness during the third trimester by ultrasound either transabdominal sonography (TAS) or transvaginal sonography (TVS). The purpose of this study was to determine the role of ultrasound in the evaluation of scar thickness for prediction of uterine dehiscence in term women with prior C-sections.

## METHODS

This retrospective cross-sectional study was conducted in the Radiology department of Aga Khan University hospital from 1^st^ July to 31^st^ December 2021. The inclusion criteria were pregnant women 18 years or above, live singleton fetus with vertex presentation, at 36+ weeks of gestation, history of prior caesarean section and availability of post-partum medical record.

### Exclusion criteria:

It comprised of multiple pregnancies, placenta previa, morbidly adherent placenta, lower uterine segment fibroid and absence of relevant medical record. The ultrasound was performed by sonographers with a minimum of five years’ experience in obstetric ultrasound. The ultrasounds were performed on Canon Xario and Aplio machines using two to five MHz convex transducer. Using the standardized technique, with optimally distended urinary bladder, the measurement for the LUS thickness was performed transabdominally.

The thickness of LUS can be measured as full thickness which includes measurement between the urine in maternal urinary bladder and the amniotic fluid. The other method is the measurement of the myometrial layer only and includes measurement of the hypoechoic part of the LUS lying between the echogenic urinary bladder wall and the chorioamniotic membrane.^7^

**Fig.1:**
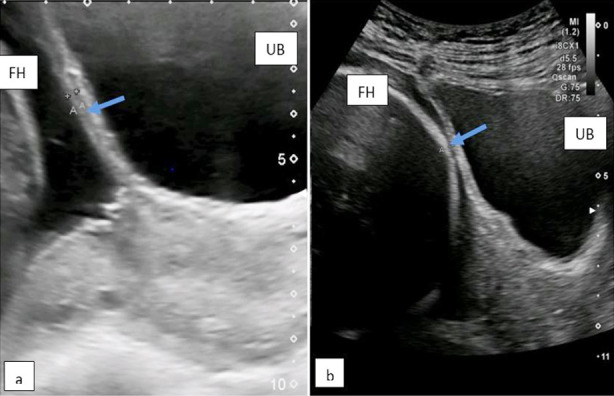
TAS showing LUS thickness of: a=2.6 mm, b=1.3 mm. Arrow at lower uterine scar (LUS) measuring myometrial thickness. UB-urinary bladder; FH-fetal head

In the present study the myometrial thickness was measured in the sagittal plane, the thinnest area of the segment was focused, and the measurements were taken with cursors at the hypoechoic myometrial layer. The image was enlarged to reduce the variation of measurement to less than 0.1mm. Three measurements were taken, and the lowest thickness was recorded. (Fig.1)

The intraoperative visual findings of the appearance of LUS at the time of C-section were recorded and categorized into two groups for statistical analysis. Group I included a well-developed lower uterine segment, and thin lower uterine segment with non-visualization of contents and group II comprised of translucent lower uterine segment /dehiscence with visualization of contents.

### Ethical Approval:

The study protocol was approved by the Ethic committee of the University (Date 01-Aug-2020, number 2020-3616-11439).

### Statistical Analysis:

Data was analyzed using STATA version 16.0. The distribution of the quantitative variables such as age, gestational age, and scar thickness were assessed using the Shapiro wilk test. Mean ±standard deviation was calculated and reported for all normally distributed quantitative variables. Median (IQR) was calculated and reported for all non-normally distributed quantitative variables. Frequencies and percentages were calculated for all categorical variables. The association of the quantitative independent variables with the outcome was assessed using t-test and Mann-Whitney U test.

Similarly, the association of the categorical independent variables with the outcome was assessed using chi-square and fisher exact test and a p-value of <0.05 was considered significant. Scar thickness was categorized at ultrasound scan according to different cutoffs reported in the literature. A 2×2 contingency table was constructed to measure sensitivity, specificity, positive, negative predictive value, and area under the curve (AUC) for each cutoff using receiver operating characteristic (ROC) analysis.

## RESULTS

One hundred and twenty-six women were included in the study. The mean age of the study participants was 29.8 ±4.1. The median gestational age was 35 (34-37) weeks. The median LUS thickness was 3.6 (2.8-4.2) millimeters. Most of the study participants 78 (61.9%) had history of one prior C-section. The inter pregnancy interval (IPI) of three years was seen in 33 (26.4%) women, followed by two years in 29 (23.2%). In terms of comorbidities, most of the study participants i.e., 103 (80.5%) did not report any comorbidities, followed by gestational diabetes mellitus (GDM) with 10 (7.8%) patients reporting it.

In the majority of the patients i.e., 119 (94.4%), the LUS measurement was done at the time of routine growth scan, and only in a few cases 07(5.6%) lower abdominal pain was the indication for LUS thickness determination. All patients underwent repeated C-section and the main indication was a prior C-section found in 114 (90.5%) of the patients, followed by failed VBAC 09 (7.1%) and fetal distress 03 (2.4%). (Table-I).

**Table-I:** Patient Demographics.

Variable	N (%)
Age	29.8 ±4.1
Gestational Age (weeks)	35 (34-37)
Scar Thickness	3.6 (2.8-4.2)
***Number of Previous C-sections***	
1	78 (61.9%)
2	25 (19.8%)
3	15 (11.9%)
4 or more	08 (06.3%)
***Interpregnancy Interval***	
1	14 (11.2%)
2	29 (23.2%)
3	33 (26.4%)
4	22 (17.6%)
5 or more	27 (21.6%)
***Comorbid***	
None	103 (80.5%)
GDM	10 (7.8%)
PIH/Preeclampsia	05 (3.9%)
Other	10 (7.8%)
***Indication of USG***	
Growth Scan	119 (94.4%)
Lower Abdominal Pain	07 (5.6%)
***Indication for C-section***	
Previous Scar	114 (90.5%)
Failed VBAC	09 (7.1%)
Fetal Distress	03 (2.4%)

Among the study groups i.e., with dehiscence and without dehiscence, the age and gestational ages were similar in both the groups and were statistically insignificant (p=0.08 & p=0.94) respectively. No difference was detected in the scar thickness of the patients among both the groups with a median scar thickness of 3.7mm and 3.5mm respectively (p=0.1). The three years IPI was the commonest among both the groups with 30 (26.3%) and 03 (27.3%) of the patients, though not found to be statistically significant (p=0.2).

A significant association was found between number of previous C-sections and the outcome with number of well-formed lower segments decreasing among those who had more c-sections (p=0.01) (Table-II). Similarly, among the maternal comorbidities, Gestational diabetes mellitus (GDM) showed a significant association (p=0.05). The sensitivity, specificity, positive predictive value, negative predictive and area under the curve (AUC) values for the different cutoffs that have been reported in the literature. (Table-III)

**Table-II:** Patient Demographics among Study Groups 126 (115+11).

Variable	Well Formed Lower Segment/Thinned Out (contents not visible) 115(91.3%)	Scar Dehiscence (contents visible) 11 (8.7%)	P-value
Age	30.04 ±3.9	27.8 ±5.3	0.08^Ŧ^
Gestational Age (weeks)	35 (34-37)	36 (33-37)	0.9^¶^
***Number of Previous C-sections***			
1	72 (62.6%)	06 (54.5%)	0.01^‡^
2	25 (21.7%)	0 (0.0%)	
3	13 (11.3%)	02 (18.2%)	
4 or more	05 (4.3%)	03 (27.3%)
***Interpregnancy Interval***			
1	12 (10.5%)	02 (18.2%)	0.2^‡^
2	26 (22.8%)	03 (27.3%)	
3	30 (26.3%)	03 (27.3%)	
4	19 (16.7%)	03 (27.3%)	
5 or more	27 (23.7%)	0 (0.0%)	
Scar Thickness	3.7 (2.8-4.3)	3.5 (2.1-4.0)	0.1^¶^
***Comorbid***			
None	96 (83.5%)	07 (63.6%)	0.4^§^
GDM	07 (6.1%)	03 (27.3%)	0.05^§^
PIH/Preeclampsia	04 (3.5%)	01 (9.1%)	1.00^§^
Other	09 (7.8%)	01 (9.1%)	1.00^§^
***Indication of USG***			
Growth Scan	109 (94.8%)	10 (90.9%)	0.4^‡^
Lower Abdominal Pain	06 (5.2%)	01 (9.1%)
***Indication for C-section***			
Previous Scar	103 (89.6%)	11 (100%)	1.00^‡^
Failed VBAC	09 (7.8%)	0 (0.0%)	
Fetal Distress	03 (2.6%)	0 (0.0%)	

Ŧ T-test, ¶ Mann-Whitney U test, § Chi-Square, ‡ Fisher Exact.

**Table-III:** Diagnostic Accuracy measures for Different Cutoffs.

Lower Uterine Segment	Sensitivity	Specificity	PPV	NPV	AUC
≤2	93%	18.2%	18.2%	93%	0.55
≤2.5	80.9%	36.4%	36.3%	80.8%	0.58
≤3	70.4%	45.4%	45.4%	70.4%	0.57
≤3.5	54.8%	54.5%	54.5%	54.7%	0.54

**Fig.2:**
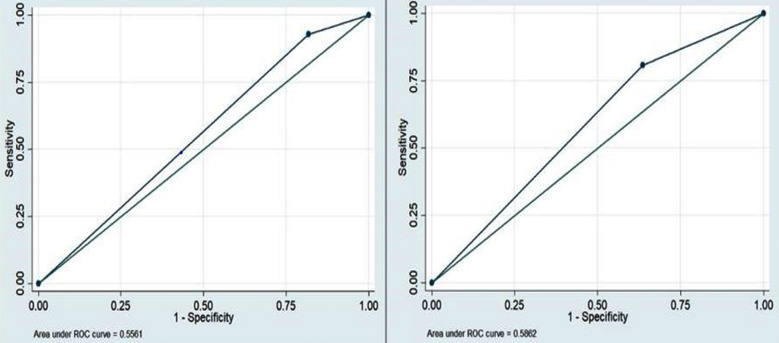
AUC for the cut off value of < 2 and < 2.5 mm.

The highest AUC 0.58 was recorded for the scar thickness of ≤2.5mm with a sensitivity, specificity, PPV and NPV of 80.9%, 36.4%, 36.3% and 80.8% respectively. Similarly, the AUC for the scar thickness of ≤ 2mm was 0.55 with a sensitivity, specificity, PPV and NPV of 93%, 18.2%, 18.2% and 93% respectively (Fig.2).

## DISCUSSION

Over the past few decades, the rate of CD has significantly increased, and one of the many medical and non-medical reasons that contribute to the fall of vaginal birth after CD is the danger of uterine dehiscence and rupture, which can result in serious maternal and perinatal complications. In the present study uterine dehiscence was seen in 8.7% of the study participants, and similar results were reported by Uharček P et al.^8^ A randomized multicenter controlled trial,^9^ conducted to determine the effects of recommending a method of delivery based on ultrasound assessment of LUS thickness, showed uterine dehiscence rate of 1.0% in the study group and 1.2% in the control group. The relatively high percentage of dehiscence in the present study could be because participants with more than one C-section were also included. However, Sarwar et al.^10^ reports, 33% of study subjects having thin or dehiscent scar at the time of CD.

Most of the patients’ demographics and clinical data in the present study showed no significant association with the outcome, i.e., scar dehiscence, consistent with the observation reported by Sarwar et al.^10^ There was no difference in the scar thickness of the patients among both groups i.e., with and without dehiscence with a median scar thickness of 3.5mm and 3.7 mm respectively (p=0.1). The two variables in the present study that showed an association were the number of previous C-sections (p=0.01) and gestational diabetes mellitus (GDM p=0.05). Other studies however have reported an association of clinical features like maternal age, IPI, method of surgical site closure and infection at surgical site with risk of scar dehiscence and rupture.^11^,^12^ Thus the utility of clinical criteria to independently forecast the likelihood of LUS dehiscence or uterine rupture should be used with caution.

Sharma et al.^13^ included full thickness TAS measurement of LUS with a cutoff value of 3.65 mm and reported a sensitivity of 91 %, specificity 93 %, and negative predictive value 91 %. It has been reported that the outer bladder wall unlikely contributes to the functional integrity of the LUS. Hence measuring the myometrial thickness alone will be a more accurate indicator of the integrity of LUS and numerous authors have shown its connection to uterine scar defects.^14^,^15^ In the present study only, the myometrial thickness was measured hence similar conclusions can be drawn.

Several studies have reported that the thinning of the LUS on ultrasound in third trimester in women with previous CD strongly predicts the presence of a defective scar.^6^,^9^ However, because the optimal cutoff values and the best US technique have not been verified, the sonographic LUS examination is infrequently carried out in clinical practice. Cutoff values suggested in earlier studies range from 1.4 to 2.0 mm for the myometrial layer and from 2.0 to 3.5 mm for the overall LUS thickness.^16^ The result of the present study showed the highest sensitivity (93%) and NPV (93%) for the LUS thickness of < 2.0mm. However, the highest AUC (0.58) was noted for the LUS thickness measurement at a cut off value of < 2.5mm.

It is important that the method of ultrasound measurement is communicated clearly to the obstetrician in the ultrasound report as there are different cutoff values reported for both methods. The LUS can be measured via TAS, TVS or as a combination of both. Martins et al.^17^ reported transvaginal measurement of LUS to be more reliable while Sen et al.^18^ reported excellent correlation between per abdominal and transvaginal measurement of LUS thickness. In the present study, the measurement of LUS was done via TAS due to our referring physician’s preference.

An important strength of the study is that it clearly mentions the methodology used for measurement of LUS i.e., the myometrial layer. This study’s high NPV for the critical thickness suggests that a thick LUS is often strong. The LUS thickness in the present study was measured in patients who were not in labor, and it is postulated that the fetal head descent during labor may stretch the LUS and make it thinner.

### Limitations:

It is a single center retrospective study. The AUC of the data ranged from 0.54 to 0.58, these values could have been enhanced by increasing the sample size.

## CONCLUSION

Ultrasound evaluation of LUS thickness is valuable approach to assess the scar integrity, it correlates with scar dehiscence and can provide guidelines for obstetricians with regards to delivery options in women with prior C-sections. A cutoff value of < 2mm showed a high sensitivity and negative predictive value of 93% for the risk of uterine dehiscence.

Due to the heterogeneity of literature on this topic and the different cut off values for the risk of uterine dehiscence it’s important that the ultrasound report clearly mentions the technique of US i.e. TAS or TVS and whether the LUS measurements include full thickness or only the myometrial layer. The US measurements, however, should be used in conjunction with other clinical findings and feto-maternal risks.

### Author`s Contribution:

**SA:** Conception, designing, drafting and revision of the article. and final approval of the version submitted.

**IM:** Conception, drafting and revision of the article. Review and final approval of the version submitted.

**AA:** Data analysis and interpretation, drafting of methodology and results, review and approval of final version.

**AM:** Conception, study design, acquisition of data, and revising it critically. review and final approval of the version submitted.

All authors agree to be accountable for all aspects of the work and questions related to the accuracy or integrity of any part of the work.

## References

[1] Weerasinghe K, Rishard M, Brabaharan S, Walpita Y (2023). Physiotherapy training and education prior to elective Caesarean section and its impact on post-natal quality of life:a secondary analysis of a randomized controlled trial. BMC Research Notes.

[2] Boerma T, Ronsmans C, Melesse DY, Barros AJ, Barros FC, Juan L (2018). Global epidemiology of use of and disparities in caesarean sections. Lancet.

[3] Keag OE, Norman JE, Stock SJ (2018). Long-term risks and benefits associated with cesarean delivery for mother, baby, and subsequent pregnancies:Systematic review and meta-analysis. PLoS Med.

[4] Salmanian B, Fox KA, Arian SE, Erfani H, Clark SL, Aagaard KM (2020). In vitro fertilization as an independent risk factor for placenta accreta spectrum. Am J Obstet Gynecol.

[5] Zhang H, Haiyan LI, Shouling LU, Weirong GU (2021). Oxytocin use in trial of labor after cesarean and its relationship with risk of uterine rupture in women with one previous cesarean section:a meta-analysis of observational studies. BMC Pregnancy Childbirth.

[6] Swift BE, Shah PS, Farine D (2019). Sonographic lower uterine segment thickness after prior cesarean section to predict uterine rupture:A systematic review and meta-analysis. Acta Obstet Gynecol Scand.

[7] Cheung VY, Constantinescu OC, Ahluwalia BS (2004). Sonographic evaluation of the lower uterine segment in patients with previous cesarean delivery. J Ultrasound Med.

[8] Uharček P, Brešťanský A, Ravinger J, Máňová A, Zajacová M (2015). Sonographic assessment of lower uterine segment thickness at term in women with previous cesarean delivery. Arch Gynecol Obstet.

[9] Rozenberg P, Sénat MV, Deruelle P, Winer N, Simon E, Ville Y (2022). Evaluation of the usefulness of ultrasound measurement of the lower uterine segment before delivery of women with a prior cesarean delivery:a randomized trial. Am J Obstet Gynecol.

[10] Sarwar I, Akram F, Khan A, Malik S, Islam A, Khan K (2020). Validity of transabdominal ultrasound scan in the prediction of uterine scar thickness. J Ayub Med Coll Abbottabad.

[11] Eleje GU, Udigwe GO, Okafor CG, Njoku TK, Okoro CC, Onyejiaka CC (2023). Intra-operative Diagnosis of Lower Segment Scar Dehiscence in a Second Gravida After One Previous Lower Segment Cesarean Section:Should We Advocate for Routine Antenatal Uterine Scar Thickness Testing?. Clin Med Insights Case Rep.

[12] Tazion S, Hafeez M, Manzoor R, Rana T (2018). Ultrasound Predictability of Lower Uterine Segment Cesarean Section Scar Thickness. J Coll Physicians Surg Pak.

[13] Sharma C, Surya M, Soni A, Soni PK, Verma A, Verma S (2015). Sonographic prediction of scar dehiscence in women with previous cesarean section. J Obstet Gynaecol India.

[14] Jastrow N, Chaillet N, Roberge S, Morency AM, Lacasse Y, Bujold E (2010). Sonographic lower uterine segment thickness and risk of uterine scar defect:a systematic review. J Obstet Gynaecol Can.

[15] Seliger G, Chaoui K, Lautenschläger C, Riemer M, Tchirikov M (2018). Technique of sonographic assessment of lower uterine segment in women with previous cesarean delivery:a prospective, pre/intraoperative comparative ultrasound study. Arch Gynecol Obstet.

[16] Gizzo S, Zambon A, Saccardi C, Patrelli TS, Di Gangi S, Carrozzini M (2013). Effective anatomical and functional status of the lower uterine segment at term:estimating the risk of uterine dehiscence by ultrasound. Fertil Steril.

[17] Martins WP, Barra DA, Gallarreta FM, Nastri CO, Filho FM (2009). Lower uterine segment thickness measurement in pregnant women with previous Cesarean section:reliability analysis using two-and three-dimensional transabdominal and transvaginal ultrasound. Ultrasound Obstet Gynecol.

[18] Sen S, Malik S, Salhan S (2004). Ultrasonographic evaluation of lower uterine segment thickness in patients of previous cesarean section. Int J Gynaecol Obstet.

